# Predicting and characterizing selective multiple drug treatments for metabolic diseases and cancer

**DOI:** 10.1186/1752-0509-6-115

**Published:** 2012-08-29

**Authors:** Giuseppe Facchetti, Mattia Zampieri, Claudio Altafini

**Affiliations:** 1 Statistical and Biological Physics Department, SISSA (International School for Advanced Studies), Via Bonomea 265 - 34136, Trieste, Italy; 2 Institute of Molecular Systems Biology, ETH (Eidgenoessische Technische Hochschule), Wolfgang Pauli Str. 16 - 8093, Zurich, Switzerland; 3 Functional Analysis DepartmentSISSA (International School for Advanced Studies), , Via Bonomea 265 - 34136, Trieste, Italy

**Keywords:** Metabolic network, Drug synergism, Flux balance analysis, Metabolic diseases, Cancer

## Abstract

**Background:**

In the field of drug discovery, assessing the potential of multidrug therapies is a difficult task because of the combinatorial complexity (both theoretical and experimental) and because of the requirements on the selectivity of the therapy. To cope with this problem, we have developed a novel method for the systematic in silico investigation of synergistic effects of currently available drugs on genome-scale metabolic networks.

**Results:**

The algorithm finds the optimal combination of drugs which guarantees the inhibition of an objective function, while minimizing the side effect on the other cellular processes. Two different applications are considered: finding drug synergisms for human metabolic diseases (like diabetes, obesity and hypertension) and finding antitumoral drug combinations with minimal side effect on the normal human cell. The results we obtain are consistent with some of the available therapeutic indications and predict new multiple drug treatments. A cluster analysis on all possible interactions among the currently available drugs indicates a limited variety on the metabolic targets for the approved drugs.

**Conclusion:**

The in silico prediction of drug synergisms can represent an important tool for the repurposing of drugs in a realistic perspective which considers also the selectivity of the therapy. Moreover, for a more profitable exploitation of drug-drug interactions, we have shown that also experimental drugs which have a different mechanism of action can be reconsider as potential ingredients of new multicompound therapeutic indications. Needless to say the clues provided by a computational study like ours need in any case to be thoroughly evaluated experimentally.

## Background

In spite of the advances in molecular and computational biology, the discovery of new drugs still remains a very challenging task which requires a very long period of research and development before any new compound can be commercialized. A possible alternative to the search of new active compounds is to make use of the unexploited properties of already available drugs, since a wide knowledge about both their therapeutic and toxicity effects has already been gathered during the study for their approval. In this perspective, a natural approach to broaden the range of applications of the existing drugs is to try to combine them in multiple drug therapies [1-3]. However, even though both the financial burden of conducting trials as well as the risk of adverse events in trial populations is expected to be sensibly lower for already approved drugs, so far the experimental investigations of multicomponent therapies have been quite limited [4,5]. Major obstacles to this approach are the high number of possible combinations but also our limited understanding of the complex mode of action of a multidrug treatment. Indeed, multiple perturbations can show three types of interaction, which have been classified as synergistic, antagonistic and additive [6] (alternatively called, aggravating, buffering and non-epistatic [7]). We have focused our attention on the first type, where the use of drug combinations represents an enhancement with respect to the superposition of the single perturbations.

In order to identify synergistic effects, Ref. [4] investigated all pairs of a set of known drugs at different doses, obtaining a functional classification of the interactions by looking at their inhibitory effect. Various computational approaches, based on reconstructed genome-scale metabolic networks, have been also developed in Refs. [7-10], in order to identify the synergistic effects triggered by multiple drugs or multiple genetic perturbations (for example the so-called “synthetic lethality”). Unfortunately, a systematic evaluation of the effects of all possible combinations of drugs is unfeasible, because their number scales exponentially with the number of chemicals taken into account (for an exhaustive search over 40 drugs, more than a trillion possible combinations should be tested). Moreover, a drug profile is given by both its therapeutic effect and by its side effect, the latter being related to its selectivity. For example, drugs such as anticancer agents have to selectively act only on tumor cells [11]. Similarly, metabolic diseases are induced by the imbalance of key metabolic pathways which, if modulated without affecting other vital functions, can rescue from the pathology [12,13]. Referring to these specific needs, we have developed an algorithm based on the metabolic network of humans and on the comparison between the metabolic networks of human and cancer cells (as reconstructed recently in [14]) aimed at expanding the spectrum of applications of the existing drugs to new selective treatments against metabolic diseases and tumors.

**Figure 1:**
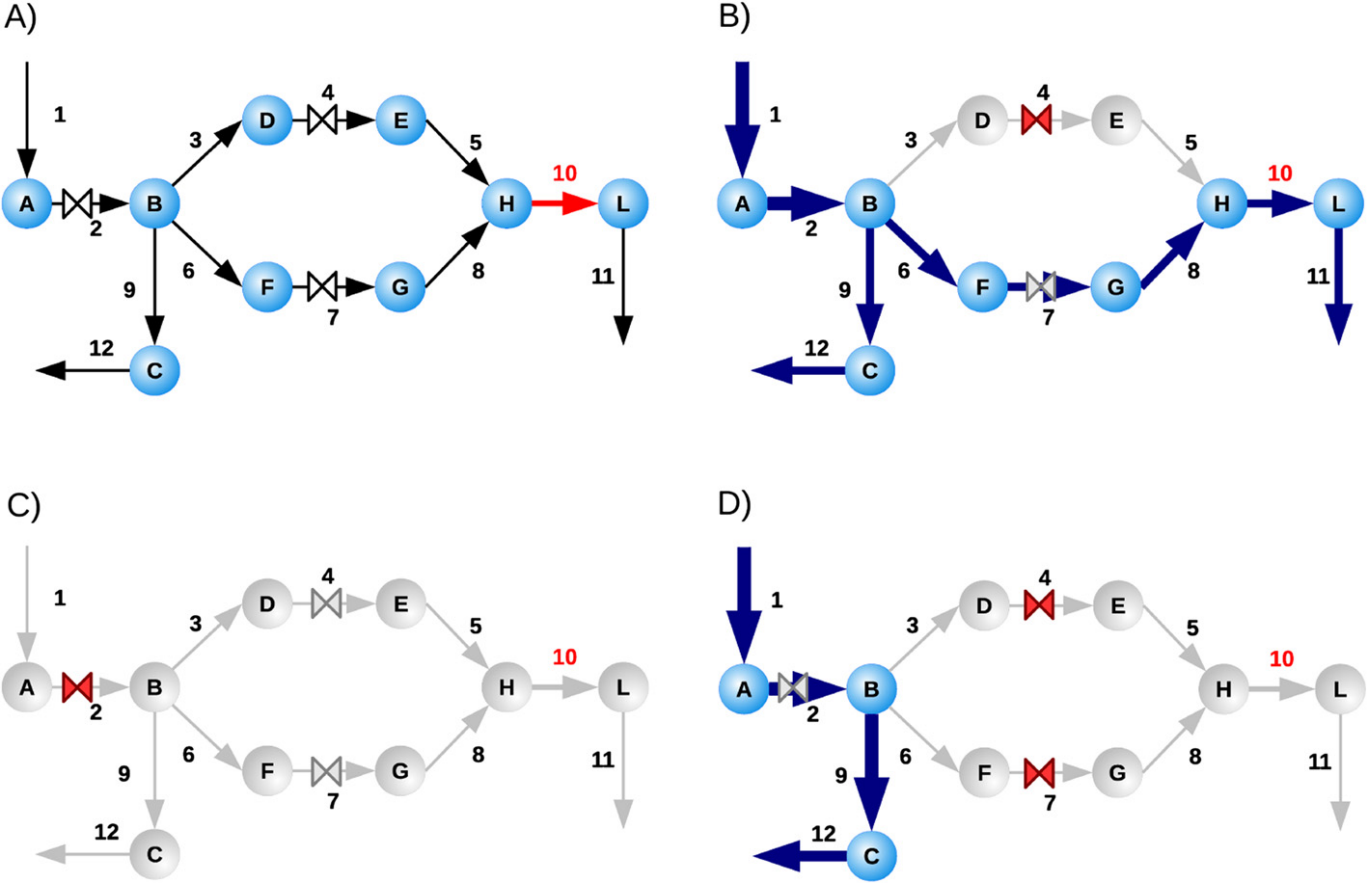
**Example of drug synergism in FBA.** For the toy network depicted in (**A**) the aim is to stop the objective reaction *v*_10_ (in red) by choosing a combination of drugs (the three valves “⋈”) while blocking the minimum number of reactions other than *v*_10_. In the drawing, blue arrows indicate active fluxes while gray arrows refer to stopped reactions; the valve is red if the drug is used, gray otherwise. Panel (**B**) shows how the use of a single drug (*v*_4_ or *v*_7_) does not stop the objective reaction, while the drug at *v*_2_ blocks the objective reaction *v*_10_ but it also blocks all fluxes of the network (panel (**C**)). Therefore, the optimal drug combination blocking the objective function *v*_10_ with minimal side effect is given by the synergism of the two drugs acting at *v*_4_ and *v*_7_ (panel (**D**)). The comparison of panels **(B)** and **(D)** shows how a synergism is a behavior which cannot be simply inferred by the superposition of the effects of the single drugs, but that structurally depends from the topology of the network.

The algorithm here proposed is based on Flux Balance Analysis (FBA) [15,16]: in spite of its simple formalism, FBA has already proven to be reliable in providing quantitative understanding of cell metabolism. The computational method presented in this paper is based on a bilevel optimization which, after reformulation through duality theory, allows the algorithm to efficiently search the interactions between drugs. With respect to the available literature [7-9,17-19], the procedure we are proposing presents at least three important differences: (i) the synergisms are efficiently explored over all drug combinations without limiting only to pairwise combinations but without doing an exhaustive search, thanks to the application of duality theory; (ii) the multiple drug treatments suggested by the method guarantee both the inhibition of the chosen target (efficacy) and a minimal side effect on the other cellular functions (selectivity); (iii) in our procedure, any metabolic process of the network can be chosen as possible disease and phenotype readout, not only cell growth as common in the FBA literature (a more detailed comparison with the current literature is reported in the Additional file 1). Inspired by works such as Refs. [20,21], we treat the inhibition of a metabolic reaction by a drug as the silencing of the gene which codes for the catalyzing enzyme. In this simplified framework, the synergistic effect resulting from multiple perturbations of the metabolic network is still well captured [7,22] (see Figure 1 for a simple example). The selectivity of any drug treatment is correlated to its side effect, which is estimated as number of stopped reactions (see Figure 1 (C) _*obj*_*vs* (D)) plus a correction term for the known non-metabolic targets. Further details are described in the Methods Section. It is worth noting that, because of the steady state assumption of FBA, this formalism does not identify synergisms between drugs that manifest themselves as alterations of kinetic parameters and consequently of concentration of metabolites, like the case of drugs which act on the same linear path (as e.g. Trimethoprim and Sulfamethoxazole do on folate synthesis [23]). Similarly, interactions where for instance one drug inhibits the biodegradation of the others cannot be found by FBA-based methods [24]. 

In spite of the limitations of restricting our analysis only to metabolic networks, the results presented in the following provide a promising example of how *in silico* models can be used as practical tools for exploring the functional interactions between drugs and of the (little explored) potential offered by synergistic drug combinations.

## Results and discussion

In order to explore its potentialities and limitations, the algorithm (described in the Methods Section) has been applied to two different case studies: 

1. finding drug synergisms for metabolic diseases (like diabetes, obesity and hypertension) on the human network [25];

2. finding antitumoral drug combinations with minimal side effect on the normal human cell (using the cancer network of [14] to model the metabolism of a human tumor).

Some features of these two metabolic networks are listed in Table 1, together with the number of drugs currently approved (from [26]). In particular, in order to generate realistic solutions, the available information about these existing drugs has been carefully filtered, selecting only inhibitions of metabolic human targets that have been experimentally proven (more details in Methods, Additional file 1: Table S1 and Additional file 2). 

**Table 1:** Features of the metabolic networks considered in the paper

	**Human [**[25]**]**	**Cancer [**[14]**]**
Number of reactions	2469	940
Number of metabolites	1587	654
Number of compartments	8	8
Number of pathways	83	62
Number of drugs	85	55

The human network has been obtained from BIGG (http://bigg.ucsd.edu/), whereas the cancer network has been provided us by the authors of [14]. The number of reactions here reported is before the splitting of every reversible process in a pair of irreversible reactions. Drugs have been selected from DrugBank database [26] as described in Methods. The complete list is reported in Additional file 1: Table S1).

### Human metabolism

In this Section, we consider the inhibition of specific functions of the human metabolism obtained without impairing other vital processes. First, the inhibitory effect of each single drug on the whole network has been calculated. Then, the following screening is performed: we systematically consider each reaction of the network as a potential objective function and we apply the algorithm, searching for the most selective synergism capable of blocking this objective reaction. Comparing this solution with the single-drug effects evaluated in advance, we distinguish three cases: 

the drug combination leads to a *new inhibition* since no single drug can stop the objective reaction;

the objective reaction can be stopped also by a single drug but the drug combination is *more selective* (has a minor side effect);

the objective reaction can be stopped also by a single drug and the multiple drug solution is *less selective* (this solution is not interesting because it triggers a larger side effect).

**Figure 2:**
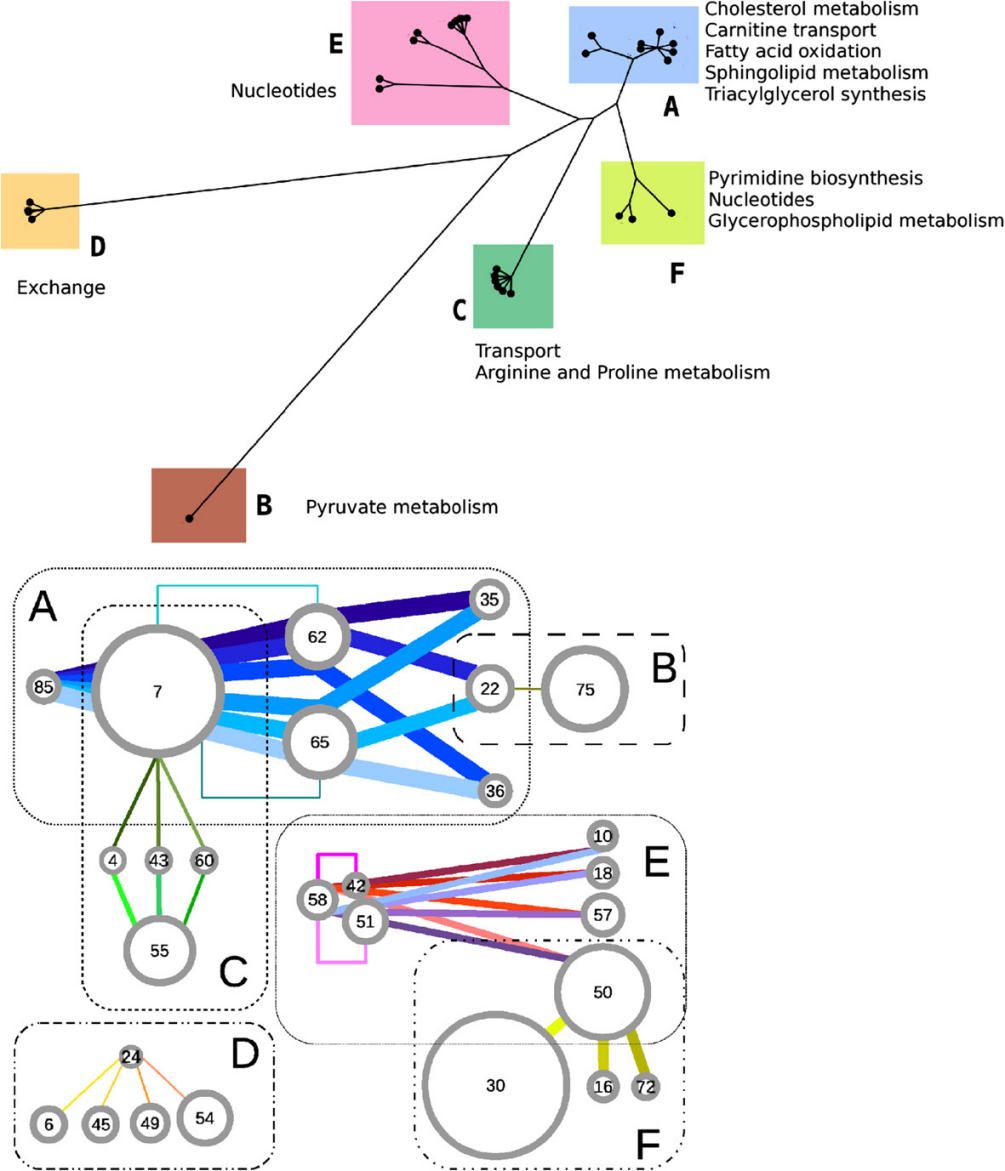
**Classification of the synergisms for the human metabolic network. ***Top panel*: Each leaf of the tree represents a multidrug solution that we have found. The layout of the graph is obtained through the same method used for phylogenetic trees (a distance tree, see text) and manifestly shows the clustering of these synergisms; the six clearly visible classes have been labeled with letters (from “**A**” to “**F**”). Names of the pathways mainly affected by each class are reported near the clusters. *Bottom panel*: This network of drugs represents a detailed characterization of the classes of synergisms. Each drug is indicated by a circle (whose radius is proportional to the number of its direct targets; drugs are labeled with numbers according to Additional file 1: Table S1). Each synergism is drawn as a colored line which connects the drugs involved (each synergism has its own color and the line thickness is proportional to the number of stopped reactions). Even in this more detailed representation, the six classes are still visible. Some subclasses can also be identified: drug pairs (7, 62) and (7, 65) in class A and drug pairs (42, 58) and (51, 58) in class E (indicated with broken lines) exploit part of the synergism of the entire class; indeed these 4 pairs are the isolated leaves in the corresponding clusters in the top panel. Note the role of drugs 7, 22 and 50 in bridging classes **A**-**C**, **A**-**B** and **E**-**F**. Details of the metabolic functions to which these classes of synergisms correspond are given in Figure 3.

In all cases where a single or a multidrug solution is found, also all suboptimal solutions are hierarchically identified, iterating the procedure while excluding the current optimum, until the problem becomes unfeasible (i.e. no more solutions exist, capable of blocking that objective reaction). At the end of the screening, we obtained a set of 32 multicomponent solutions, ranging from combinations of two up to four compounds (see Table 2). The following characterization of the synergistic effects is performed. For each combination we identify the set *Y * of metabolic reactions which cannot be stopped by any single drug of the combination, but which are stopped when all these drugs are used together. Then, the synergism is described by the vector $\left(\text{s}\in {\left\{0,1\right\}}^{N_{r}}\right)$, where *s*_*j*_ = 1 if reaction *j* belongs to *Y * (*N*_*r*_ is the number of reactions in the metabolic network). From the vectors **s** of the 32 multiple drug solutions a matrix of distances can be constructed and a cluster analysis performed on these distances; the resulting distance-based tree (similar to a phylogenetic tree) is drawn in Figure 2 (upper panel). The synergisms are clustered in six classes (with clearly identifiable subclasses in some of them) labeled from “A” to “F”. This classification can be used to build also a proximity network for the drugs, linking those that belong to the same synergistic interaction. The outcome is drawn in Figure 2 (bottom panel) and shows that the same clustering applies to the drugs involved in the synergisms. The result highlights how drugs can often be used in alternative one to the others: for example, the synergistic pairs of class C contain one drug among those labeled with the number 7 or 55 (Rosiglitazone or Acetylsalicylic acid) in combination with one drug among number 4 or 43 or 60 (Pravastatin or Naftifine or Tioconazole; see Additional file 1: Table S1 for all correspondences between names and numbers). Being the cardinality of class C equal to 6, we can deduce that these solutions are generated only by the combination of the pair and the triplet just mentioned. Three exceptions to the sharp clusterization of Figure 2 are represented by drugs labeled with the numbers 7, 22 and 50, respectively Rosiglitazone, Indomethacin and Pentoxifylline. Indeed Rosiglitazone targets many metabolic reactions (60, all in the fatty acid metabolism) which allow two types of interaction: class A for fatty acid activation and class C for cholesterol metabolism. On the other hand, Indomethacin causes only 7 inhibitions, some belonging to glycerolphospholipids metabolism and some others to pyruvate pathways: the first interact synergistically with drugs which target fatty acid reactions (class A), whereas the second can be combined with drugs acting on pyruvate metabolism (like Fomepizole, drug number 75, in class B). Finally, Pentoxifylline inhibits reactions both in the salvage pathway for nucleotides (which give synergisms in class E) and in pyrimidine catabolism (class F). 

**Table 2:** List of all drug synergisms

**Drugs**	**Side eff.**	**Syn.**	**ratio**	**Class**
**Rosiglitazone** (*#*7) - Quinacrine (*#*36) - **Cerulenin** (*#*62) - Tyloxapol (*#*85)	363.8	91	25.0%	A
Rosiglitazone (*#*7) - Quinacrine (*#*36) - Orlistat (*#*65) - Tyloxapol (*#*85)	377.7	91	24.0%	A
**Rosiglitazone** (***#***7) - Indomethacin (*#*22) - **Cerulenin** (*#*62) - Tyloxapol (*#*85)	390.6	91	23.2%	A
**Rosiglitazone** (*#*7) - Diclofenac (*#*35) - **Cerulenin** (*#*62) - Tyloxapol (*#*85)	397.5	91	22.8%	A
Rosiglitazone (*#*7) - Indomethacin (*#*22) - Orlistat (*#*65) - Tyloxapol (*#*85)	404.5	91	22.4%	A
Rosiglitazone (*#*7) - Diclofenac (*#*35) - Orlistat (*#*65) - Tyloxapol (*#*85)	411.4	91	22.1%	A
**Rosiglitazone** (***#***7) - **Cerulenin** (***#***62)	298.9	52	17.3%	A
Rosiglitazone (*#*7) - Orlistat (*#*65) -	312.8	52	16.6%	A
Indomethacin (*#*22) - Fomepizole (*#*75)	84.7	1	1.1%	B
Naftifine (*#*43) - Acetylsalicylic acid (*#*55)	116.0	6	5.1%	C
Acetylsalicylic acid (*#*55) - Tioconazole (*#*60)	116.0	6	5.1%	C
**Simvastatin/Pravastatin** (*#*4) - **Acetylsalicylic acid** (*#*55)	123.9	6	4.8%	C
Rosiglitazone (*#*7) - Tioconazole (*#*60)	280.9	6	2.1%	C
Rosiglitazone (*#*7) - Naftifine (*#*43)	280.9	6	2.1%	C
**Simvastatin/Pravastatin** (*#*4) - **Rosiglitazone** (*#*7)	288.8	6	2.0%	C
Carbidopa (*#*6) - Droxidopa (*#*24)	93.1	1	1.0%	D
Droxidopa (*#*24) - Selegiline (*#*45)	96.1	1	1.0%	D
Droxidopa (*#*24) - Minaprine (*#*49)	152.4	1	0.6%	D
Droxidopa (*#*24) - Zonisamide (*#*54)	289.7	1	0.3%	D
Mycophenolic acid (*#*42) - Mercaptopurine (*#*58)	11.0	5	45.4%	E
Ribavirin (*#*51) - Mercaptopurine (*#*58)	23.9	5	20.9%	E
Udenafil (*#*10) - Mycophenolic acid (*#*42) - Mercaptopurine (*#*58) -	18.0	7	38.8%	E
Mycophenolic acid (*#*42) - Dipyridamole (*#*57) - Mercaptopurine (*#*58)	22.0	7	31.8%	E
Udenafil (*#*10) - Ribavirin (*#*51) - Mercaptopurine (*#*58)	30.9	7	22.6%	E
Ribavirin (*#*51) - Dipyridamole (*#*57) - Mercaptopurine (*#*58)	34.9	7	20,0%	E
Theophylline (*#*18) - Mycophenolic acid (*#*42) - Mercaptopurine (*#*58)	41.7	7	16.7%	E
Mycophenolic acid (*#*42) - Pentoxifylline (*#*50) - Mercaptopurine (*#*58)	53.8	7	13.0%	E
Theophylline (*#*18) - Ribavirin (*#*51) - Mercaptopurine (*#*58)	54.6	6	10.9%	E
Pentoxifylline (*#*50) - Ribavirin (*#*51) - Mercaptopurine (*#*58)	66.7	6	8.9%	E
Pentoxifylline (*#*50) - Arsenic trioxide (*#*72)	118.2	17	14.3%	F
Cladribirne (*#*16) - Pentoxifylline (*#*50)	118.2	17	14.3%	F
Gemcitabine (*#*30) - Pentoxifylline (*#*50)	157.8	15	9.5%	F

The table reports the multiple drug solutions in the human metabolic network, the side effect *σ*(*D*), the synergism size (i.e. the number of stopped reactions which exceeds the linear superposition of single drug effects), the ratio between these two quantities, and their classification (see Figure 2 and main text for the clustering analysis). Drug numbers refer to Additional file 1: Table S1. Bold font indicates that the solution (or part of it) has an experimental validation.

**Figure 3:**
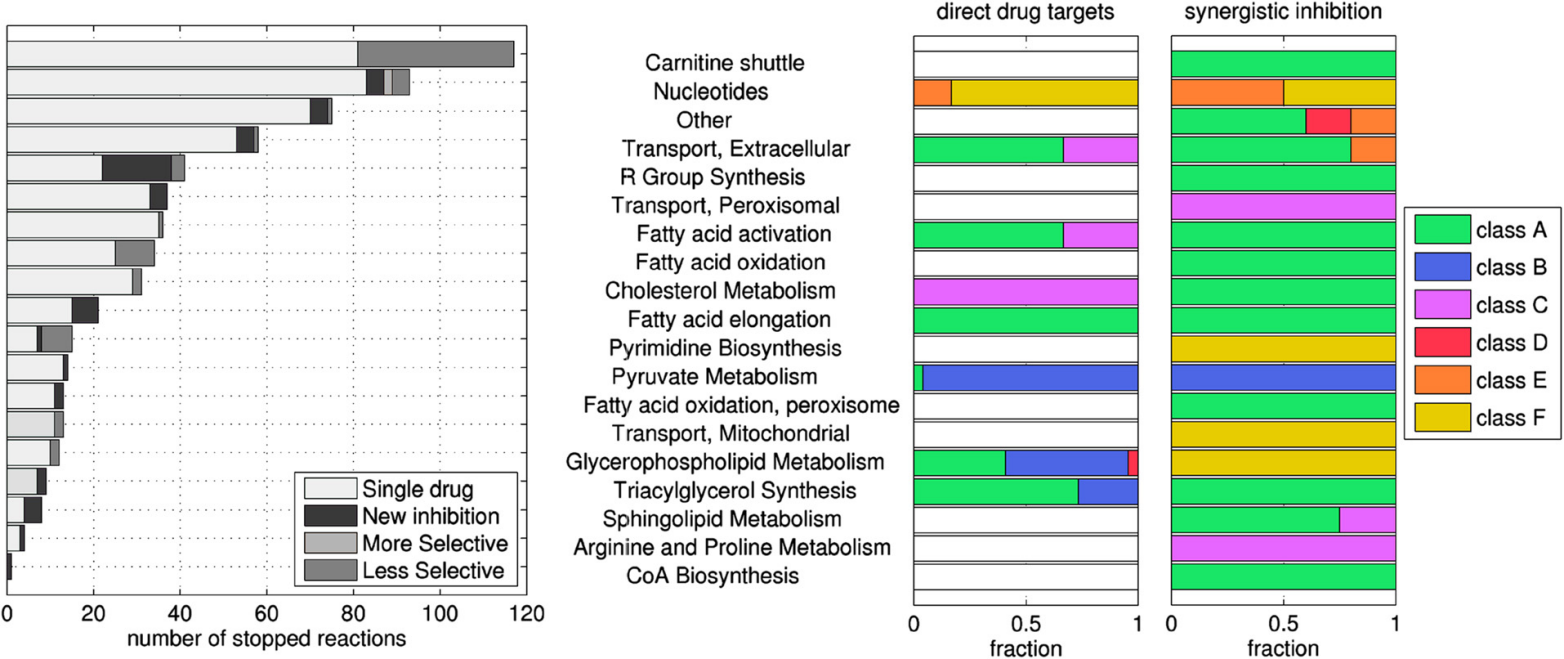
**Drug synergisms for the human metabolic network. ***Left panel*: For each affected pathway, the histogram reports the number of objective reactions which can be stopped; gray-scale bars represent reactions stopped only by a *Single drug* or multidrug solution, classified as *New inhibition* (meaning that no single drug is capable of triggering the inhibition), *More selective* and *Less selective* inhibitions (referring to the case where both single and multiple drug treatments are possible and the multiple one has respectively a lower and a higher side effect). *Right panels*: The two plots refer to multiple drug solutions only. For the same pathways as in the left panel, we report here the fraction of the direct drug targets and the fraction of the synergistic inhibitions which are induced by the six classes of synergisms (shown in Figure 2): the comparison between the two stacks shows that synergistic interactions can occur on pathways that are not direct targets of the drugs.

The analysis of the complete results obtained from the screening over all metabolic reactions is shown in Figure 3, where the stoppable reactions are grouped on the basis of the metabolic pathway to which they belong. Figure 3 reports also the class and the targets of the drug combinations which induces the inhibition. As one can see, some synergisms occur between reactions which belong to different pathways: in particular, sphingolipids subsystems, CoA and pyrimidine biosynthesis contain reactions whose inhibitions are caused by interactions situated in other pathways, since none of the combined drugs have targets on them. Moreover, among the multiple drug solutions of Figure 3, there are several *new inhibitions* and a few *more selective* cases (a comparison of the side effects induced by single and multiple drug solutions is reported in Additional file 1: Figure S1). 

**Figure 4:**
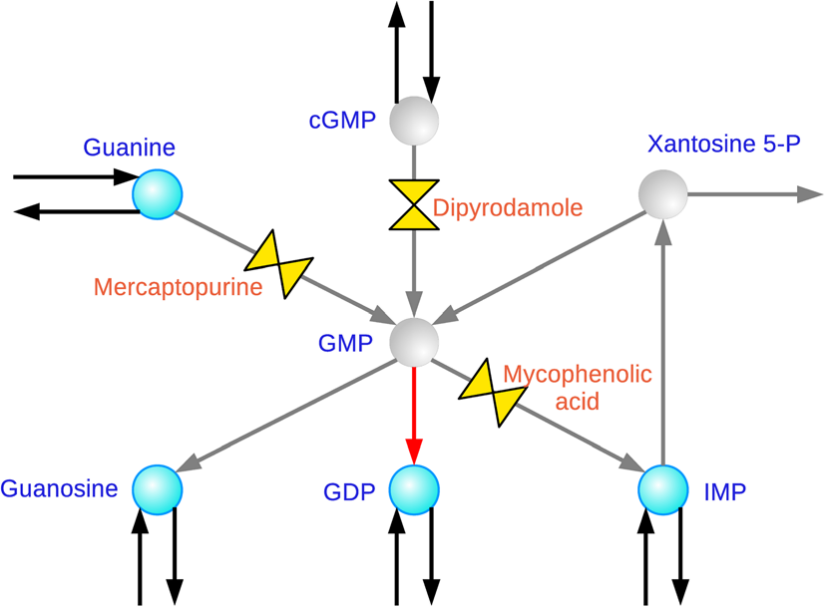
**Nonlinearity in the synergism: the example of Guanylate kinase.** The part of the human network here represented shows the nonlinear interaction when the three drug targets (the three valves, with the name of the drugs) are simultaneously inhibited: when this is the case, the objective reaction of Guanylate kinase (in red) is stopped. Gray arrows and gray circles indicate respectively stopped reactions and metabolites which become unavailable.

Among the cases of new inhibitions, the case of Guanylate kinase, although of no therapeutic interest, represents an easily visualizable example of the nonlinearity in the superposition of the effects as anticipated in the hypothetical situation presented in Figure 1. We consider the phosphorylation of GMP into GDP catalyzed by guanylate kinase as objective reaction. Since the blockage of GMP production will cause also the arrest of any transcription process, this inhibition constitutes only a toy example of synergism devoid of any practical value. For this problem, the algorithm proposes the combination of Mercaptopurine, Dipyridamole and Mycophenolic acid: the synergism takes place through the simultaneous inhibition of guanine phosphoribosyltransferase, 3^*′*^,5^*′*^-cyclic-nucleotide phosphodiesterase and IMP dehydrogenase (see Figure 4 for a representation of the corresponding subnetwork). Indeed, these reactions are alternative ways of GMP biosynthesis. When and only when they are all blocked, Guanylate kinase lacks its substrate and stops as well. 

The complete list of the objective reactions, with relative pathways and synergistic inhibitions, is reported in Additional file 1: Table S2: this list contains many inhibitions in the fatty acid, cholesterol and carnitine transport pathways, which may represent solutions for obesity. In particular, concerning the case of hyperlipidemia diseases, the algorithm finds the combination of Rosiglitazone and Cerulenin (class A) as inhibitor of many reactions in the carnitine transferase and fatty acid desaturase pathways. This synergism has been reported in the literature for being active versus the biosynthesis of fatty acids in prostate tumors [27]: the mean IC_50_ are 45*μ*M and 32*μ*M for Rosiglitazone and Cerulenin alone, whereas it reduces to 5*μ*M when they are combined: the authors claimed that this effect comes from the reduced production of fatty acids preventing the growth and differentiation of prostate cells. It is worth noting that we predict this combination also in three anticancer solutions (together with an additional target on palmitate conversion, see next subsection). Moreover, this pair is part of other six synergisms on the human metabolism, all concerning the same pathways (i.e. a very similar mechanism of interaction).

Another significant example is represented by the inhibition of Dihydroceramide desaturase. Indeed, ceramide is the hydrophobic membrane anchor of sphingolipids and is involved as a bioactive molecule in cell growth regulation, apoptosis, senescence, and diverse cell responses, particularly those linked to stress situations [28,29]; moreover, recent studies have shown the role for ceramide biosynthesis in body weight regulation, energy expenditure, hence in the metabolic obesity syndrome [30]. For these reasons Dihydroceramide desaturase has been proposed as a promising potential target for metabolic diseases. Currently some specific inhibitors of Dihydroceramide desaturase are under investigation and development (for example GT11, XM462 and analogous [31,32]) although there is no approved drug yet. Indeed in our model no single drug can stop this reaction. Our algorithm finds some possible multidrug treatments that block this reaction: among them, there are the synergistic pairs of Rosiglitazone plus Simvastatin (Pravastatin), and Acetylsalicyclic acid plus Atorvastatin (Pravastatin). Concerning the first synergism, clinical experiments have shown that combining these two drugs a significant reduction (about 30% less) of the intracellular accumulation of lipid is achieved [33]. Moreover, the authors of [34] investigate the adverse effect of single and combined therapies (hypoglycemia, body weight increase) and claim that adverse events are generally similar (the safety profile of Rosiglitazone was not adversely affected by the addition of Atorvastatin). Also our results predict a limited worsening of the adverse effect: indeed, after the combination with Atorvastatin the side effect of Rosiglitazone passes from 252.7 (Additional file 1: Table S1) to 288.8 (Table 2), i.e. it increases of about 14% only. For the same therapeutic purpose, the pair of Acetylsalicyclic acid and Atorvastatin has been also studied. Clinical trials are currently ongoing [35] and some of them have already shown promising results [36]. The rationale for this approach is based on the restoration of platelet sensitivity by reduction of the cholesterol levels. Moreover, as mentioned above, it is known that ceramide is involved in apoptosis. Indeed, this combination has been tested for the treatment of prostate cancer: the results have shows a linear synergism between these two drugs [37].

### Human *vs* Cancer

A similar approach can be used to improve the *selectivity* and *specificity* of the treatment when dealing simultaneously with more than one type of cells. With a small adjustment, our procedure can force the solution to preserve the metabolism of one cell while inhibiting an objective reaction of another (see Additional file 1 for a detailed formulation). Indeed, a drug interaction can explore the differences in the topologies of the metabolic networks and, in this way, bypass the restrictions caused for instance by targets homology. This is crucial in case of anticancer therapy since tumoral and normal cells share the same genes.

**Figure 5:**
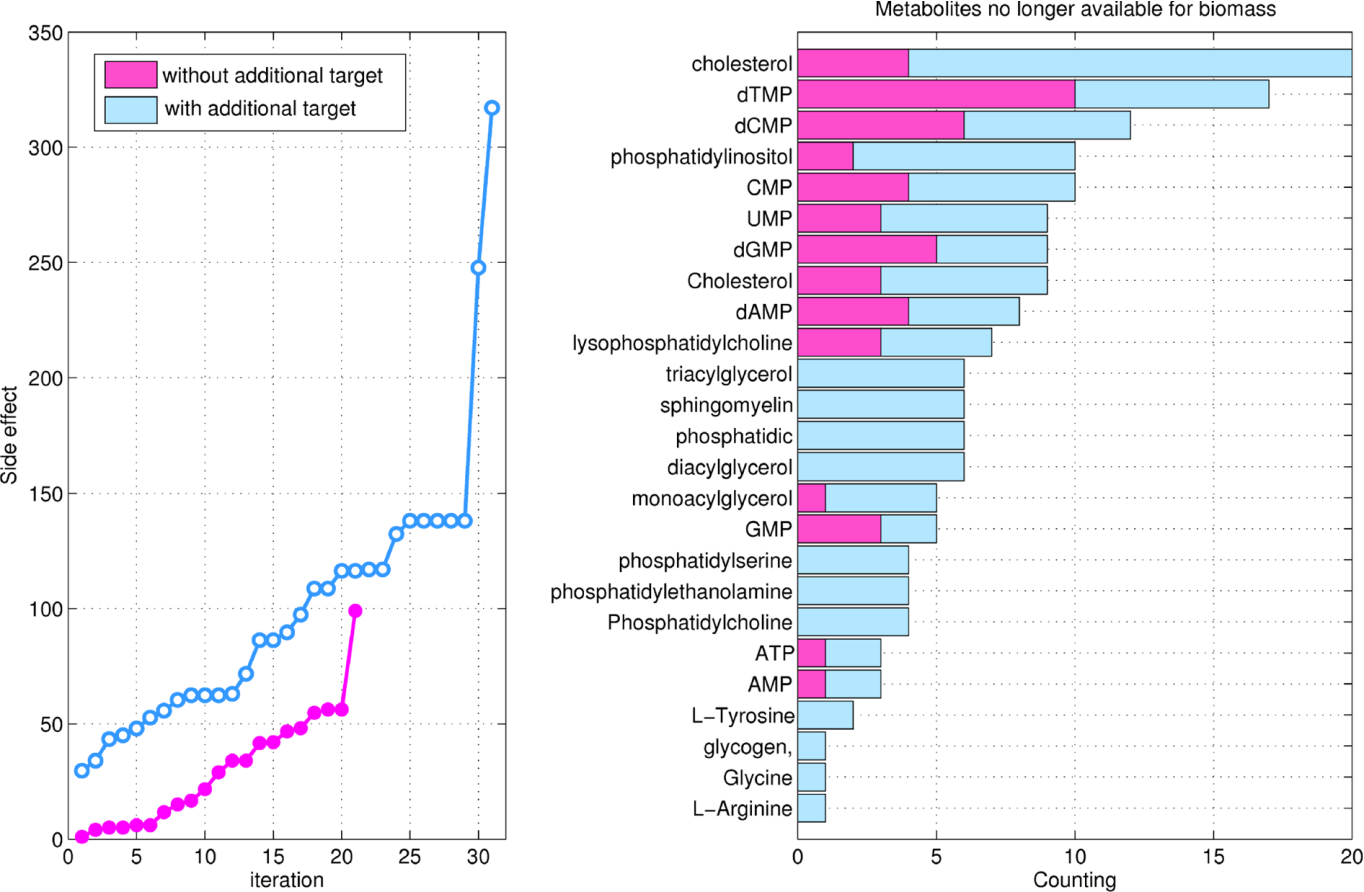
**Results on cancer *vs *human selectivity problem. ***Left panel*: The iterative application of the algorithm to the cancer *vs* human networks finds 21 solutions before becoming unfeasible (magenta line). Including the possibility of inhibiting an additional target, other 31 solutions are found (blue line). *Right panel*: The bars count the number of solutions which stop the biomass metabolite in the cancer metabolism (same color code). Solutions have been classified according to the necessity or less of the inhibition of an additional target (see Tables 3, Additional file 3: Table S3 for more details).

For this purpose, we use the metabolic network of a generic human cancer assembled in [14] and the human metabolic network. We apply the modified version of the algorithm to the biomass reaction of the cancer network (which must be stopped), while minimizing the side effect on the regular human metabolism. As in the previous section, the procedure is iterated until the problem becomes unfeasible. The results are shown in Figure 5 and Table 3. The solutions are mainly single drugs which differ one from the other in terms of side effect on the human network. Many are known chemotherapeutic agents such as Floxuridine, Mycophenolic acid, Methotrexate, Pemetrexed, Ribavirin, Myo-Inositol, Simvastatin, Leflunomide, Indomethacin, Hydroxyurea, Arsenic trioxide, Gemcitabine [38-49]. Since only one synergism is present, between Fomepizole and Auranofin, these results suggest that approved drugs do not seem to induce significant interactions at the level of the metabolism. 

**Table 3:** **Solutions for the cancer** ***vs*** **human problem**

***#***	**Drugs**	**Side effect**
1	**Floxuridine** (*#*20)	1
2	**Mycophenolic acid** (*#*42)	4
3	Trimethoprim (*#*29)	5
4	**Methotrexate** (***#***11)	5
5	Atovaquone (*#*69)	6
6	Tyloxapol (*#*85)	6
7	Ezetimibe (*#*56)	12
8	**Pemetrexed** (*#*41)	15
9	**Ribavirin** (*#*51)	17
10	Quinacrine (*#*36)	22
11	**Myo-Inositol** (*#*82)	29
12	Tioconazole (*#*60)	34
13	Naftifine (*#*43)	34
14	**Simvastatin** (*#*4)	42
15	**Leflunomide** (*#*66)	42
16	Auranofin (*#*59) - Fomepizole (*#*75)	47
17	**Indomethacin** (*#*22)	48
18	Diclofenac (*#*35)	55
19	**Hydroxyurea** (*#*16)	56
20	**Arsenic trioxide** (*#*72)	56
21	**Gemcitabine** (*#*30)	99

We report the solutions and the side effects *σ*(*D*) for the inhibition with approved drugs. Drug numbers refer to Additional file 1: Table S1. Bold font indicates that the solution has an experimental validation.

In order to increase the range of putative synergisms, we have tried to use the algorithm to set up a search for targets potentially interacting with the currently available drugs. Instead of searching for the reactions which are synthetically lethal (as partially done, for instance, in [14]) and proposing them as potential new targets, we look for the reactions whose single inhibition may give a lethal synergism with any combination of the approved drugs. The problem is formally equivalent to the one we have already described: we search again for the optimal drug combination after having deleted a reaction in the cancer network (the same reaction is removed also from the human network; this reaction is called “additional target” since it will be the target of an additional new drug). We systematically consider each reaction of the cancer network as an additional target; the results of this screening are reported in Additional file 3: Table S3. After a search in literature of possible inhibitors of the additional targets of the results, we identify some interesting solutions. For instance, the inhibition of methenyltetrahydrofolate cyclohydrolase (combined with the use of Mimosine) can be induced by the experimental drug 5,6,7,8- tetrahydro-N^5^,N^10^- carbonylfolic acid [50]; therefore, its combination with Mimosine could represent a potential antitumor therapy. Also for other four additional targets there exist experimental inhibitors whose activity is reported in the literature [51-53] (details are shown in Additional file 3: Table S3). Concerning the drug solutions identified by the algorithm, the pair Rosiglitazone plus Cerulenin is proposed in combination with three possible additional targets. One is the palmitate fatty acid conversion: it is worth noting that the validated antitumoral activity of this pairs versus prostate cancer cells (as mentioned above) is due to the reduction of the synthesis of fatty acids [27]. In our prediction, indeed, among the metabolites which are no longer available because of this inhibition, there are cholesterol ester, mono-, di- and tri-acylglycerol. The other two additional targets are related to phosphatidylcholine. Part of the phosphatidyilcholine pathway has been already identified as synthetic lethal [14], but without mentioning any possible exploitation. Our results suggest the use of a combined drug therapy as possible way to take advantage of this synthetic lethality.

## Conclusions

The field of drug combinatorics is largely unexplored experimentally and the potential of combined drug therapies is difficult to assess, mostly for lack of suitable systematic methodologies. To try to fill this gap, we have developed an algorithm which is capable of exploring efficiently the optimal synergisms among all possible drug combinations and of characterizing them in terms of side effect and selectivity. Indeed, the success of a drug discovery process depends on multiple aspects, not least on the fulfillment of requirements regarding selectivity and toxicity: for metabolic diseases the modulation of the key pathways without affecting the other vital functions can be instrumental for rescuing from the pathology. Similarly, anticancer compounds should only kill cancer cells without affecting normal ones. These requirements are rarely taken into account in standard computational approaches. The results we obtained by applying our algorithm to the human and tumor *vs* human metabolic networks show the possibility to take advantage of drug synergisms in proposing new therapies: the potentialities lay in the possibility to intervene with a different mechanism of action with respect to those that are currently available. In this enlarged repertoire of possibilities, we have identified examples of drug repurposing (some of them were previously demonstrated experimentally), a procedure which is becoming more and more attractive thanks to the reduced costs on the preclinical and clinical steps.

One of the main features of FBA-based knockout studies is that metabolic networks appear to be robust [1,17], meaning that there seem to be an high degree of redundancy of the pathways inside a network (property alternatively reported as “nonessentiality” of the gene in Refs. [8,19]). In the context of drug synergism, this property reflects into the presence of optimal solutions consisting of many drugs (in our case up to four, or even more if we consider the number of inhibition targets of each drug). For the same reason, the results show the necessity to extend the search to all possible drug combinations without limiting to those of low cardinality. This fact becomes significant especially when the drugs to combine present a high similarity in terms of inhibited targets; indeed, the characterization of the synergisms we have found shows a limited variety of possible interactions between the available drugs (only six classes were identified, see Figure 2). However, beside increasing the cardinality of the solutions, a very strong robustness may also reduce the total number of solutions because it makes more difficult to induce the simultaneous inhibition of all the redundant pathways; indeed, if the screening we have run on the human metabolism is applied also to the less robust cancer network (seen as stand-alone network), the number of possible inhibitions is much higher (see Additional file 1: Figure S2 in comparison with Figure 3). Moreover, when we want to stop the biomass reaction of the cancer, mainly single drug solutions are found: this is again an index of the low redundancy of the cancer network (see Table 3) and of the limited variety in the metabolic targets for the available drugs.

Nevertheless, adding the possibility to inhibit an extra target, we could identified some experimental compounds (other than the drugs from DrugBank) which may be used as anticancer in a combination with the approved drugs (see Additional file 3: Table S3). These examples show that predictive tools like the method we are proposing become more important if one considers also the possibility of combining active compounds which are not yet approved but for which a minimal characterization of the mechanism of action is available. In this perspective, experimental compounds which inhibit additional targets that are different from those affected by the approved drugs may represent a good chance for improving, through synergism, the spectrum of the whole set of the currently available drugs. Moreover, the application of our method can be extended to situations where multiple networks are compared and contrasted. It is expected that problems like this will become important as soon as tissue-specific networks of human metabolism and cancer-specific networks will become available in the near future.

In a broader perspective, if instead of confining our study only to the human network we consider also the metabolism of microorganisms, the exploitation of drug synergisms obtained with our algorithm can be useful in investigating a wide range of situations: (i) when specific enzyme inhibitors are not currently available, multiple drug solutions could represent an example of the *reprofiling* of existing drugs for new therapeutic indications; (ii) when the target enzyme has undergone a mutation rendering ineffective the original therapy, a synergistic solution may bypass the resistance acting on other enzymes and therefore help in *fighting resistance*; (iii) when the optimal synergism has no lethal impact while the single drug solution has. This *change in lethality* can be important for instance in cases of human-hosted bacteria producing toxic by-products: in order to save the useful symbiosis with these commensal bacteria, a selective (but not lethal) inhibition of the toxic processes must be pursued [54,55].

Apart from the specific cases we have studied, the main objective of this paper is to propose an efficient method to single out drug combinations with potentially interesting therapeutic effect. Given the exponential character of the combinatorics involved, unguided “fishing expeditions” are intrinsically ill posed. Hence we expect that methods like the one proposed in this paper should be of help in dealing with such a complex problem. Needless to say, as the predictions are based on *in silico* models of metabolic pathways, the validity of the results must be assessed experimentally, but this is beyond the scope of the work.

## Methods

The drug-synergism algorithm is developed in the framework of FBA (see Supporting Information for a brief comparison with the available literature on similar methods). The problem deals with the following sets (and numbers):

$$\begin{array}{lll} \;R & =\{1,\ldots ,N_{r}\}=\text{set of reactions;}\; & \;\\ \;M & =\{1,\ldots ,N_{m}\}=\text{set of metabolites;}\; & \;\\ \;D & =\{1,\ldots ,N_{d}\}=\text{set of drugs;}\; & \;\\ \;T_{j} & =\{1,\ldots ,N_{t,j}\}=\text{set of drugs having the reaction}\; & \;\\ & \;j\;\text{as a target.}\; \end{array}$$

Then, the optimal synergism problem can be stated as follows:

**Problem:***Given:*

• *a metabolic network, which means a stoichiometric matrix*$S\in R^{N_{m}\times N_{r}}$*and a vector of fluxes ***v*** with upper-bounds ****U****, both laying in*$R^{N_{r}}$*;*

• *an objective reaction flux v_obj_ which has to be stopped;*

• *the set*D *of drugs together with their inhibition targets;*

*we want to find the subset of drugs*D⊆D*such that D blocks v*_*obj*_* causing the minimal side effect, i.e. a minimum perturbation on the overall reaction fluxes.*

Of course, we are not interested in procedures which perform an exhaustive search in the space of all drugs combinations.

By decomposing any reversible reaction in a couple of irreversible reactions, we can always assume that fluxes have non-negative values. Then, the space *H* of all possible steady state fluxes **v** as defined by FBA is

$$H:=\{v\;:\;\mathrm{Sv}=0,\;\;\;0\leq v_{j}\leq U_{j}\;\forall j\in R\},$$

where *vj* and *U*_*j*_ are the *j*-th components of the vectors **v** and **U** respectively. Given the inhibition targets of each drug, we assume that a drug inhibits completely the enzymes responsible for the targeted reactions, hence stopping the relative fluxes. Therefore, adding a drug combination *D* to the problem means forcing (directly or indirectly) to zero some of the fluxes. We introduce the binary variables ${\left\{d_{k}\right\}}_{k\in D}$ such that

$$d_{k}=\left\{\begin{array}{c} 0\;\;\text{if drug}\;k\;\text{is used (i.e.}\;k\in D);\\ 1\;\;\text{if drug}\;k\;\text{is not used (i.e.}\;k\notin D). \end{array}\right)$$

Then, if the *k*-th drug targets the *j*-th reaction we write:

$$v_{j}\leq U_{j}d_{k}.$$

This reduces the space of feasible steady state fluxes to a subset *H*(*D*) ⊂ *H* (notice that *H*(*∅*) ≡ *H*). We can now introduce a definition which quantifies the *side effect*, *σ*(*D*), of a drug combination *D* on the reaction fluxes.

Clearly, there are many possible definitions. If we were dealing with microorganisms we could adapt the analogous MOMA (Minimization of Metabolic Adjustment) or ROOM (Regulatory On/Off Minimization) [20,21] criteria modeling the perturbation effect (induced by knockout effect, in the literature) on the fluxes with respect to the “wild type” fluxes. This requires however to know the unperturbed fluxes (the wild type reference) which for a microorganism corresponds to using the biomass production as cost function of the FBA problem [56]. Unfortunately, for human metabolic network such a commonly accepted FBA criterion is unavailable, hence it is not possible to determine the metabolic fluxes for the unperturbed network (“wild type”). Consequently, also the calculation of the perturbed fluxes and the quantification of the side effect are more ambiguous. Our choice in this paper is to quantify the side effect as number of stopped reaction (more precisely the number of reactions that cannot take place because of the perturbation). This bypasses the lack of the reference fluxes and allows to quantify the number of cellular functions which are *no longer available*, regardless to the type of tissue to which the human cell belongs.

Since the available drugs we selected do not have only metabolic targets, a measure of the side effect based exclusively on metabolic reactions disregards the perturbation induced by the drugs on other cellular functions (for example signaling cascades, protein synthesis, etc). This additional information can be incorporated in the model weighting each drug variable *d*_*k*_ in the objective function according to its non-metabolic effects (for which only a pure superposition is considered because of the lack of more quantitative models). Following this approach, we define:

(1) $$\sigma (D):=\overset{N_{r}}{\underset{j=1}{\sum }}(1-y_{j})+\overset{N_{d}}{\underset{k=1}{\sum }}\beta_{k}(1-d_{k});$$

where the parameter *β*_*k*_ is an estimation of the non-metabolic perturbation induced by drug *k* and *y*_*j*_ is a binary variable such that *y*_*j*_ = 0 when the flux is lower than a threshold *ϵ* (*ϵ* = 0*.*1 in this paper); this can be expressed by means of the following linear constraints:

$$\begin{array}{lll} \;\epsilon y_{j}\leq v_{j} & \;\;\forall j\in R;\; & \;\\ U_{j}y_{j}\geq v_{j} & \;\;\forall j\in R.\; & \; \end{array}$$

In order to avoid a double count, for reversible reactions we impose the additional constraint

$$\begin{array}{lll} y_{j}+y_{l}\leq 1 & \;\forall j\;\text{and}\;l\in R\;\text{s.t.}\;v_{j}\; & \;\\ & \;\text{and}\;v_{l}\;\text{are opposite fluxes}.\; & \; \end{array}$$

Although the choice of the weights *β*_*k*_ is quite arbitrary, we have tried to evaluate both terms of (1) using a homogeneous criteria, relating the values of *β*_*k*_ to the number of non-metabolic targets of each drug. In particular we set:

$$\beta_{k}:=\bar{\beta }\cdot \text{[\# non-metabolic targets of drug}\;k],$$

where the numerical coefficient $\bar{\beta }$ captures the spread of the perturbation across the non-metabolic systems. In analogy with the metabolic network, $\bar{\beta }$ is equal to the mean number of metabolic reactions that are stopped when a single metabolic target is inhibited. Referring to the human metabolic network and averaging over all drugs we selected, we obtain $\bar{\beta }=7.7$.

The optimal solution can be described as follows:

**Solution: ***For any subset of drugs*D⊆D*, we can find the set H(D) and the minimal perturbation σ(D). By restricting to drug combinations which inhibit the objective reaction (i.e. such that*$\left({\text{max}}_{v\in H(D)}(v_{\text{obj}})=0\right)$*) the optimal solution Y is*

(2) $$Y=\arg\underset{\left[\begin{array}{c} D\subseteq D\\ {\text{max}}_{v\in H(D)}(v_{\text{obj}})=0 \end{array}\right]}{\text{min}}\sigma (D).$$

The resulting bilevel optimization is a min-max integer linear programming problem [57]. The inner problem adjusts the fluxes in order to achieve the *maximum* flow for the objective reaction when all fluxes are subjected to the inhibitions (drugs) imposed by the outer problem and to the stoichiometric constraints. The outer problem selects the combination of drugs which *minimizes* the side effect, restricting to those solutions of the inner problem which guarantee no flow for the objective reaction.

The bilevel optimization problem is the following:

$$\begin{array}{cc} \text{Minimize} & \sum_{j=1}^{N_{r}}\alpha_{j}(1-y_{j})+\sum_{k=1}^{N_{d}}\beta_{k}(1-d_{k})-b\sum_{k=1}^{N_{d}}d_{k}\;\text{“outer problem”}\\ \text{such that} & \\ & \left[\begin{array}{cccc} \text{Maximize} & v_{\text{obj}} & & \;\text{“inner problem”}\\ \text{such that} & & & \\ & \sum_{j=1}^{N_{r}}S_{i,j}v_{j}=0 & \;\forall i\in M & \\ & v_{j}\leq U_{j} & \;\forall j\in R & \\ & v_{j}\leq U_{j}d_{k} & \;\forall j\in R,k\in T_{j}; & \end{array}\;\right]\\ & v_{\text{obj}}=0;\\ & \epsilon y_{j}\leq v_{j}\;\forall j\in R;\\ & U_{j}y_{j}\geq v_{j}\;\forall j\in R;\\ & y_{j}+y_{l}\leq 1\;\forall j\in R\;\text{such that}\;v_{l}\text{is the opposite flux of}\;v_{j}, \end{array}$$

where the parameter *b* ≪ 1 (*b* = 0*.*001 in this paper) is introduced in the objective function of the outer problem in order to exclude the combinations containing redundant inhibitions and therefore avoiding an “over-selection” of drugs.

To solve this bilevel optimization we apply the strong duality theorem which consists in appending a list of constraints corresponding to the dual of the inner problem and setting the primal objective function equal to the dual [58,59]. This leads to a single minimization problem. By calling the dual variables as follows

$$\begin{array}{lll} \;\mu_{1},\ldots ,\mu_{N_{m}}\in R: & \text{associated to the first}\;N_{m}\; & \;\\ & \text{constraints in the inner problem};\; & \;\\ \lambda_{1},\ldots ,\lambda_{N_{r}}\in R_{+}: & \text{associated to the second set of the}\; & \;\\ & \text{constraints of the inner problem};\; & \;\\ \;\delta_{1},\ldots ,\delta_{N_{t}}\in R_{+}: & \text{associated to drugs targets (third}\; & \;\\ & \text{set of constraints;}\;N_{t}:=\;\overset{N_{r}}{\underset{j=1}{\sum }}\;N_{t,j}),\; & \; \end{array}$$

the final optimization problem becomes:

Minimize

$$\overset{N_{r}}{\underset{j=1}{\sum }}\alpha_{j}(1-y_{j})+\;\overset{N_{d}}{\underset{k=1}{\sum }}\beta_{k}(1-d_{k})-b\overset{N_{d}}{\underset{k=1}{\sum }}d_{k}$$

such that

$$\begin{array}{lcr} \overset{N_{r}}{\underset{j=1}{\sum }}S_{i,j}v_{j}=0 & \;\forall i\in M;\\ v_{j}\leq U_{j} & \;\forall j\in R;\\ v_{j}\leq U_{j}d_{k} & \;\forall j\in R,k\in T_{j};\\ \overset{N_{m}}{\underset{i=1}{\sum }}S_{i,j}\mu_{i}+\lambda_{j}+\overset{N_{t,j}}{\underset{i=1}{\sum }}\delta_{i}\geq 0 & \;\forall j\in R\setminus \{\text{obj}\};\\ \overset{N_{m}}{\underset{i=1}{\sum }}S_{i,j}\mu_{i}+\lambda_{j}+\;\overset{N_{t,j}}{\underset{i=1}{\sum }}\delta_{i}\geq 1; & \;\text{for}\;j=\text{obj}; & \end{array}$$

(3) $$\begin{array}{lc} v_{\text{obj}}=\overset{N_{r}}{\underset{i=1}{\sum }}U_{i}\left(\lambda_{i}+\delta_{i}\underset{k\in T_{i}}{\sum }d_{k}\right); & \\ v_{\text{obj}}=0; & \\ \epsilon y_{j}\leq v_{j} & \;\forall j\in R;\\ U_{j}y_{j}\geq v_{j} & \;\forall \;j\;\in R;\\ y_{j}+y_{l}\leq 1 & \;\forall j\;\in \;R\;\text{such that}\;v_{l}\;\text{is}\\ & \;\text{the opposite flux of}\;v_{j}. \end{array}$$

The key simplification is that the nonlinear terms *δ*_*i*_*d*_*k*_ =: *z*_*ik*_ in (3) (the strong duality theorem equality) are exactly linearizable as follows:

(4) $$0\leq z_{\mathrm{ik}}\leq \delta_{i}^{\max}d_{k}$$

(5) $$\delta_{i}-\delta_{i}^{\max}(1-d_{k})\leq z_{\mathrm{ik}}\leq \delta_{i}$$

where $\left(\delta_{i}^{\max}\right)$ is the upper bound for the dual variable *δ*_*i*_(chosen arbitrarily big in the implementation).

The codes for the algorithm have been developed in MATLAB (MathWorks R2010b) and are available in Additional files 4, 5, 6, 7, 8 and 9. All Mixed Integer Linear Optimizations have been performed using the ILOG-IBM CPLEX 12.1, under free academic license.

### Selection procedure for the drugs

In order to generate realistic solutions, drugs have been accurately selected from http://www.drugbank.ca[26] according to the following procedure (the output of the query is summarized in Additional file 1: Table S1): 

1. the whole database contains 6708 drugs;

2. only *approved* drugs have been picked out, restricting the search to 1570 drugs;

3. we filter for drugs which act on *human* enzymes (identified by the EC number): the set reduces to 473 drugs;

4. we select only drugs for which an *inhibitory* effect on at least one enzyme of the human metabolism has been experimentally proven. The set reduces to 267 drugs (with the EC numbers of the inhibited enzymes). For these drugs, we count the number of non-metabolic targets, including also cases of agonism, antagonism and activation, since they all represent a perturbation to the regular functioning of the target (activation is not considered for metabolic targets because it does not affect the number of stopped reaction in our FBA formulation).

5. the reactions of the metabolic network directly inhibited by each drug are identified through the available correspondence between EC numbers of the inhibited enzymes and the gene codes first, and then through the correspondence between gene codes and metabolic reactions. During this step, it may happen that many genes, and hence many reactions, are associated to the same EC number. Therefore, although in the original database a drug inhibits only a single or a few targets, the number of metabolic reactions affected by the drug can be high. For instance, this is the case of Rosiglitazone which inhibits a single target, the long-chain-fatty-acid-CoA ligase an enzyme responsible for the binding of the acyl-CoA group to a long fatty acid chain. Since the substrate of this enzyme can be any carbon chain, regardless of the unsaturation (presence or not of double bonds C=C) and of the exact length (it just requires a chain longer than 12 carbons atoms), we end up with a drug which inhibits up to 60 metabolic targets.

6. drugs which have exactly the same metabolic targets are grouped; the final 85 groups are listed in Additional file 1: Table S1. For each group only a representative is reported, namely the drug which has the minimal number of targets outside the metabolism. The full list of drugs in each group is reported in the Additional file 2.

## Abbreviations

FBA: Flux Balance Analysis; MOMA: Minimization of Metabolic Adjustment.

## Competing interests

The authors declare that they have no competing interests.

## Authors’ contributions

MZ and CA conceived and initiated the project. GF developed the method and performed the calculations. All authors participated in the discussions of the results, have been involved in drafting the manuscript and approved the final manuscript’s preparation.

## Supplementary Material

Additional file 1 **Supplementary_information.** It contains: a brief literature survey about the main computational methods in the literature, details about the algorithm for competitive organisms (cancer *vs* human), Additional file 1: Table S1 with the list of drugs selected from DrugBank database, Additional file 1: Table S2 of the reactions of human metabolism inhibited by multiple drug solutions, Additional file 1: Figure S1 for the comparison of the side effects and Additional file 1: Figure S2 about the results on cancer metabolic network alone. Click here for file

Additional file 2 **Drugs.** Excel file with the details about the results of the query on DrugBank, including the name of all drugs with the same metabolic targets (and the group representative we have chosen). Click here for file

Additional file 3 **Table S3.** Cancer *vs* human network: solutions with an additional target. Click here for file

Additional file 4 **Human.** MATLAB script for running the algorithm on the human metabolic network. Click here for file

Additional file 5 **Human_PROBLEM.** MATLAB data with the model of the human metabolic network. Click here for file

Additional file 6 **Best_drug_synergism.** MATLAB function with the implementation of the algorithm for single network problems (a short help is inside the code). Click here for file

Additional file 7 **Cancer_VS_Human.** MATLAB script for running the algorithm on cancer *vs* human network. Click here for file

Additional file 8 **Cancer_VS_Human_PROBLEMS.** MATLAB data with the information about the problem of cancer *vs* human. Click here for file

Additional file 9 **Best_drug_synergism_2.** MATLAB function with the implementation of the algorithm for competitive networks (a short help is inside the code). Click here for file
