# Supplementary material for: Predicting and characterizing selective multiple drug treatments for metabolic diseases and cancer
Source: BMC Syst Biol. 2012 Aug 29;6:115. doi: 10.1186/1752-0509-6-115 (PMC3744170; doi:10.1186/1752-0509-6-115)
Supplement: Additional file 1 — Supplementary_information. It contains: a brief literature survey about the main computational methods in the literature, details about the algorithm for competitive organisms (cancer vs human), Additional file 1: Table S1 with the list of drugs selected from DrugBank database, Additional file 1: Table S2 of the reactions of human metabolism inhibited by multiple drug solutions, Additional file 1: Figure S1 for the comparison of the side effects and Additional file 1: Figure S2 about the results on cancer metabolic network alone. [file 1752-0509-6-115-S1.pdf]

Predicting and characterizing selective multiple drug treatments for metabolic diseases and cancer: SUPPORTING INFORMATION

Giuseppe Facchetti, Mattia Zampieri, Claudio Altafini

A brief literature survey

In this Section we present a quick overview of the literature and of the main computational methods which investigate drug synergism (or synergism induced by similar network perturbations, like gene knockout). We list them from the most similar to the most different with respect to our algorithm, underlying the main discrepancies.

Optknock and OptOrf [1, 4]: the two works aim to find the best knockout (simple or multiple, up to a limit in cardinality fixed by the user) that maximizes the biosynthesis of a given metabolite while optimizing the biomass production. Both methods are based on FBA (OptOrf includes also some transcriptional regulations). The bilevel optimization contained in both algorithms has been reformulated through the duality theory, which assures the computational efficiency of the method. Indeed, we have been inspired by these works in developing of our algorithm.

Essentiality and synthetic lethality [7]: Through an algorithm which is similar to Optknock, this work identifies pairs, triple and some high-order gene deletions which are lethal in the *Escherichia coli* metabolism. Also in this method, the optimization is based on the biomass and does not consider any alternative objective reaction.

OPMET [6]: the aim is to search the gene knockout (of any cardinality) which stops a given objective reaction while inducing the minimum damage on the network (estimated as number of stopped reactions and unavailable metabolites). The approach used to model the network is similar to FBA but with a weaker assumption on the steady state: for instance, accumulation of metabolites is allowed. The search on the space of the combinations is performed dynamically through a branch-and-bound algorithm, refined with two filtering strategies. Properties of Linear Programming, like duality theory, which are useful for the efficiency of the algorithm have not been exploited.

Epistasis in human metabolism [3]: the investigation of gene epistasis has been here investigated through FBA formalism; no limitation on the cardinality of the gene deletions is imposed. However, since the search is based on an approximate determination of the elementary modes (called "pathway fragment generation"), solutions are suboptimal. An exact calculation of the elementary modes would have been computationally much more expensive and almost prohibitive for a large network such as the human. Moreover, no information on the side effect on the whole network is included.

Epistasis in yeast metabolism [5]: epistatic interactions are here studied performing an exhaustive search of all pairs of gene knockouts on the *S.cerevisiae* network modeled according to FBA; the effect is characterized through an epistasis indicator which quantifies the change of the biomass production of the multiple perturbation with respect to the two single perturbations. No objective reaction other than growth rate has been considered.

Drug targets in cancer [2]: also in this case only an exhaustive evaluation of all pairs of knockouts has been carried out; moreover, the side effect of the potential antitumoral treatments on the normal human cell is estimated in terms of ATP production, a central process in the metabolism. However, according to this evaluation criterion, all the antitumoral solutions we have found show the same impact on the human network, meaning that this definition of side effect is not able to discriminate among these different drug treatments, while our definition does. Indeed, instead of considering a single reaction, our approach estimates the impact as a global loss of the cellular functions, which in our opinion is fairly reasonable since no tissue-specific network of the human metabolism is available at the moment.

Algorithm for competitive organisms

The following version of the algorithm allows us to study the selectivity problem, in a multi-organism context, in which we would like to stop an objective reaction belonging to a first organism while having a minimal effect on a second metabolic network (whose fluxes are denoted here by $\mathbf{w}$). We assume that the two organisms live in the same environment and hence are subject to the same drugs.

$$
\begin{array}{lll}
\text{Minimize} & \sum_{i=1}^{\tilde{N}_r} \alpha_i(1-y_i) - b\sum_{k=1}^{N_d} d_k & \textit{“outer problem”} \\
\text{such that} & & \\
& \left[\begin{array}{lll}
\text{Maximize} & v_{obj} & \textit{“inner problem”} \\
\text{such that} & & \\
& \sum_{j=1}^{N_r} S_{i,j} v_j = 0; & \\
& v_j \le U_j; & \\
& v_j \le U_j d_k; &
\end{array}\right] & \\
& \sum_{h=1}^{\tilde{N}_r} \tilde{S}_{i,h} w_h = 0; & \\
& w_h \le \tilde{U}_h; & \\
& w_h \le \tilde{U}_h d_k; & \\
& v_{obj} = 0; & \\
& \varepsilon y_h \le w_h; & \\
& \tilde{U}_h y_h \ge w_h. &
\end{array}
$$

As can be seen, flux constraints ($\tilde{\mathbf{S}}\mathbf{w} = \mathbf{0}$, thermodynamical bounds $w_h \le \tilde{U}_h$ and common drugs inequalities $w_h \le \tilde{U}_h d_k$) and side effect on the second network (calculated as $\sigma(D) = \min_{\mathbf{w}} \|\mathbf{w} - \mathbf{w}^*\|$) are located only in the outer problem; therefore the fact that the side effect is now evaluated on $\mathbf{w}$ instead of on $\mathbf{v}$ does not interfer with the inner problem, i.e., with the application of the duality theorem for the first network. This separation guarantees the constraint $\max(v_{obj}) = 0$ and the optimality of the solution.

References

[1] A.P. Burgard, P. Pharkya, and C.D. Maranas. OptKnock: A bilevel programming framework for identifying gene knockout strategies for microbial strain optimization. *Biotechnol Bioeng*, 84 (6):647–657, 2003.

[2] O. Folger, L. Jerby, C. Frezza, E. Gottlieb, E. Ruppin, and T. Shlomi. Predicting selective drug targets in cancer through metabolic networks. *Mol Syst Biol*, 7, 2011.

[3] M. Imielinski and B. Belta. Deep epistasis in human metabolism. *Chaos*, 20(2):026104, 2010.

[4] J. Kim and J.L. Reed. OptORF: Optimal metabolic and regulatory perturbations for metabolic engineering of microbial strains. *BMC Syst Biol*, 4:53, 2010.

[5] D. Segré, A. De Luna, G.M. Church, and R. Kishony. Modular epistasis in yeast metabolism. *Nat Genet*, 37 (1):77–83, 2005.

[6] P. Sridhar, B. Song, T. Kahveci, and S. Ranka. Mining metabolic networks for optimal drug targets. *Pac Symp Biocomput*, 13:291–302, 2008.

[7] P.F. Suthers, A. Zomorrodi, and C.D. Maranas. Genome-scale gene/reaction essentiality and synthetic lethality analysis. *Mol Syst Biol*, 5:301, 2005.

Figures and Tables

#	Drug	M	NM	R	S.E.	cancer
1	Vigabatrin	1	1	3	17.7	X
2	Chlormerodrin	1	1	1	10.7	X
3	Fenoprofen	2	0	2	10.0	
4	Pravastatin(*)	1	1	1	34.0	X
5	Masoprocol	1	0	2	13.0	
6	Carbidopa	1	0	5	20.0	
7	Rosiglitazone	1	1	60	252.7	X
8	Zanamivir	1	0	2	8.0	
9	Papaverine	4	0	2	2.0	
10	Udenafil	1	0	3	5.0	
11	Methotrexate	1	0	2	5.0	X
12	Pantoprazole	1	0	1	1.0	
13	Methoxyflurane	3	4	1	31.8	
14	Dorzolamide	2	0	4	4.0	X
15	Diethylcarbamazine	3	0	4	23.0	
16	Hydroxyurea	4	0	4	10.0	X
17	Carmustine	1	0	2	3.0	
18	Theophylline	4	3	3	28.1	
19	Acarbose	3	1	9	26.7	
20	Floxuridine	1	0	1	1.0	X
21	Valproic Acid	2	1	5	30.7	X
22	Indomethacin	4	3	7	48.1	X
23	Nitisinone	1	0	1	5.0	
24	Droxidopa	1	9	1	70.3	X
25	Physostigmine	1	0	1	1.0	
26	Carbimazole	1	0	5	7.0	
27	Miglustat	1	0	1	5.0	
28	Allopurinol	1	0	3	11.0	
29	Trimethoprim	2	0	3	5.0	X
30	Gemcitabine	3	0	85	64.0	X
31	Acetyldigitoxin	1	0	1	1.0	X
32	Cefdinir	1	0	2	5.0	X
33	Azelaic Acid	2	1	3	14.7	
34	Pentostatin	1	0	4	4.0	X
35	Diclofenac	4	4	6	54.8	X
36	Quinacrine	2	1	4	21.7	X
37	Monobenzone	1	0	3	7.0	
38	Sulindac	3	3	9	46.1	X
39	Amodiaquine	1	0	1	5.0	
40	Tiludronate	1	1	3	13.7	X
41	Pemetrexed	4	0	8	15.0	X
42	Mycophenolic acid	2	0	1	4.0	X
43	Naftifine	1	0	1	34.0	X
44	Scopolamine	1	5	3	42.5	
45	Selegiline	2	0	5	23.0	
46	Hexachlorophene	1	1	4	9.7	X
47	Phenelzine	6	1	9	22.7	X
48	Sulfasalazine	4	3	10	55.1	X
49	Minaprine	2	7	6	77.9	
50	Pentoxifylline	4	2	21	43.4	X
51	Ribavirin	3	1	5	16.7	X
52	Phenylbutazone	3	0	2	10.0	
53	Disulfiram	2	0	9	16.0	X
54	Zonisamide	17	14	9	134.8	X
55	Acetylsalicylic acid	3	0	8	74.0	X
56	Ezetimibe	2	1	2	11.7	X
57	Dipyridamole	4	0	7	9.0	X
58	Mercaptopurine	1	0	2	2.0	X
59	Auranofin	1	1	3	10.7	X
60	Tioconazole	1	0	1	34.0	X
61	Trichlormethiazide	4	1	5	12.7	X
62	Cerulenin	4	0	7	7.0	X
63	Mimosine	2	1	5	15.7	X
64	Perhexiline	2	0	52	113.0	X
65	Orlistat	2	1	10	22.7	X
66	Leflunomide	1	3	3	42.1	X
67	Trilostane	2	2	2	18.4	
68	Aminoguanidine	1	0	2	4.0	
69	Atovaquone	3	0	1	6.0	X
70	Carvedilol	1	8	1	62.6	X
71	Desipramine	1	12	1	98.4	X
72	Arsenic trioxide	1	6	1	56.2	X
73	Cyclizine	1	1	2	10.7	
74	Dazoxiben	1	0	1	6.0	
75	Fomepizole	4	0	15	33.0	X
76	Clomipramine	1	5	4	57.5	
77	Bepridil	2	7	3	56.9	X
78	Lithium	3	2	5	46.4	X
79	Fidarestat	1	0	7	13.0	X
80	Carbenoxolone	1	0	4	2.0	X
81	Canaline	1	0	2	1.0	X
82	Myo-Inositol	2	0	1	29.0	X
83	Voglibose	1	0	4	7.0	
84	Icatibant	1	1	2	10.7	X
85	Tyloxapol	1	0	2	6.0	X

Table S1: **List of drugs selected from DrugBank database for human and cancer metabolic networks.** Selection criteria are explained in the main text. *Legend*: "M": number of metabolic targets; "NM": number of non-metabolic targets; "R": number of inhibited reactions of the human metabolic network; "S.E.": side effect;"X": the drug acts also on the cancer network; (*): Simvastatin and Atorvastatin are also present in this group (they induce the same metabolic inhibitions as Pravastatin but they have one and two non-metabolic targets respectively).

OBJECTIVE REACTION		SYNERGISM	
Name	Pathway	Type	Class.
Proline dehydrogenase	Arginine and Proline Metabolism	New	C
C160 transport into the mitochondria	Carnitine shuttle	Less	A
C161 transport into the mitochondria	Carnitine shuttle	Less	A
C180 transport into the mitochondria	Carnitine shuttle	Less	A
C181 transport into the mitochondria	Carnitine shuttle	Less	A
carnitine fatty-acyl transferase	Carnitine shuttle	Less	A
carnitine O-palmitoyltransferase	Carnitine shuttle	Less	A
carnitine O-stearoyl transferase	Carnitine shuttle	Less	A
carnitine octadecenoyl transferase	Carnitine shuttle	Less	A
carnitine transferase	Carnitine shuttle	Less	A
R group transport into the mitochondria	Carnitine shuttle	Less	A
transport into the mitochondria (carnitine)	Carnitine shuttle	Less	A
sterol O-acyltransferase (acyl-Coenzyme A: cholesterol acyltransferase) 1	Cholesterol Metabolism	Less	A
dephospho-CoA kinase	CoA Biosynthesis	New	A
3',5'-Cyclic GMP exchange	Exchange	New	E
(R)-Pantothenate exchange	Exchange	New	A
Ceramide 1-phosphate exchange	Exchange	New	A
cholesterol ester exchange	Exchange	Less	A
L-Phenylalanine exchange	Exchange	New	D
Beta oxidation of fatty acid	Fatty acid oxidation	Less	A
Beta oxidation of long chain fatty acid	Fatty acid oxidation, peroxisome	New	A
Beta oxidation of long chain fatty acid	Fatty acid oxidation	Less	A
fatty acyl-CoA desaturase (n-C18:1CoA -> n-C18:2CoA)	Fatty acid elongation	New	A
stearoyl-CoA desaturase (n-C18:0CoA -> n-C18:1CoA)	Fatty acid elongation	New	A
choline phosphatase	Glycerophospholipid Metabolism	Less	F
phosphatidate cytidylyltransferase	Glycerophospholipid Metabolism	Less	F
5'-nucleotidase (GMP)	Nucleotides	More	E
CTP synthase (NH3)	Nucleotides	Less	F
cytidine kinase (ATP)	Nucleotides	New	F
cytidylate kinase (CMP),mitochondrial	Nucleotides	Less	F
GMP reductase	Nucleotides	New	E
guanylate cyclase	Nucleotides	New	E
guanylate kinase (GMP:ATP)	Nucleotides	New	E
nucleoside-diphosphate kinase (ATP:CDP), mitochondrial	Nucleotides	Less	F
ribonucleoside-diphosphate reductase (GDP)	Nucleotides	More	E
aspartate carbamoyltransferase (reversible)	Pyrimidine Biosynthesis	Less	F
carbamoyl-phosphate synthase (glutamine-hydrolysing)	Pyrimidine Biosynthesis	Less	F
CTP synthase (glutamine)	Pyrimidine Biosynthesis	Less	F
dihydoorotic acid dehydrogenase (quinone10)	Pyrimidine Biosynthesis	Less	F
dihydroorotase	Pyrimidine Biosynthesis	Less	F
orotate phosphoribosyltransferase	Pyrimidine Biosynthesis	Less	F
orotidine-5'-phosphate decarboxylase	Pyrimidine Biosynthesis	Less	F
hydroxyacylglutathione hydrolase	Pyruvate Metabolism	New	B
R group artificial flux	R Group Synthesis	New	A
R group to palmitate conversion	R Group Synthesis	Less	A
R total flux	R Group Synthesis	New	A
3-Dehydrosphinganine reductase	Sphingolipid Metabolism	New	A
Ceramide kinase	Sphingolipid Metabolism	New	A
dihydroceramide desaturase	Sphingolipid Metabolism	New	C
serine C-palmitoyltransferase	Sphingolipid Metabolism	New	A
ATP transporter, peroxisomal	Transport, Peroxisomal	New	C
cGMP transport (ATP-dependent)	Transport, Extracellular	New	E
cholesterol ester transporter	Transport, Extracellular	Less	A
crmp hs transport	Transport, Extracellular	New	A
cytidine facilated transport in mitochondria	Transport, Mitochondrial	Less	F
Diphosphate transporter, peroxisome	Transport, Peroxisomal	New	C
fatty acid retinol efflux	Transport, Extracellular	New	A
intracellular transport	Transport, Mitochondrial	Less	F
NADP transporter, peroxisome	Transport, Peroxisomal	New	C
NADPH transporter, peroxisome	Transport, Peroxisomal	New	C
Pantothenate sodium symporter II	Transport, Extracellular	New	A
1-acylglycerol-3-phosphate O-acyltransferase 1	Triacylglycerol Synthesis	New	A
glycerol-3-phosphate acyltransferase	Triacylglycerol Synthesis	New	A

Table S2: **List of the inhibitions on human metabolism obtained by multiple drug solutions.** The reactions are sorted according to the pathway they belong. The solutions are classified as *New* inhibition, *More* selective and *Less* selective from a comparison with a possible single drug treatment; this information is reported in the third column. Last columns reports the class of the synergistic combination which cause the inhibition of the objective reaction (see Figure 2 on the paper).

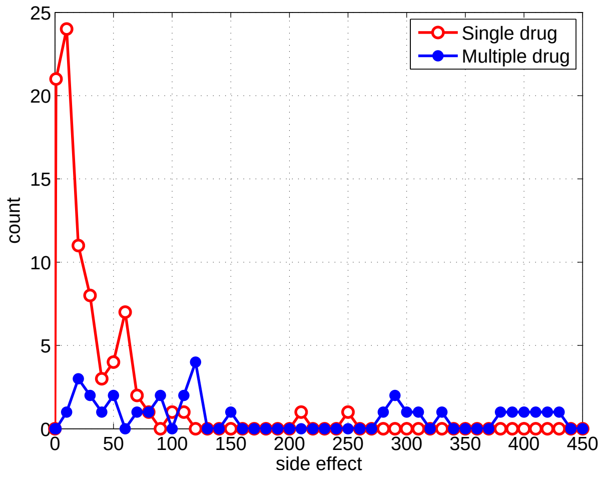

Figure S1: **Side effects comparison.** The histogram analysis of the size effect induced by single and multiple drug solutions shows that both types of solution have more or less the same order of magnitude.

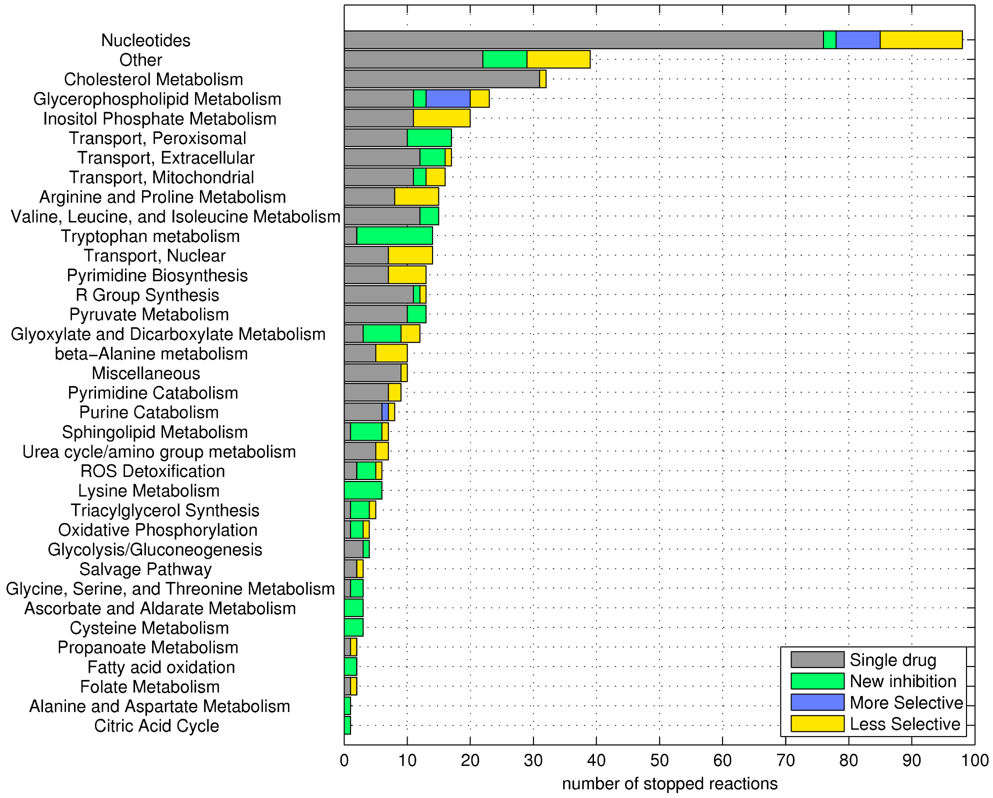

Figure S2: **Results on cancer metabolic network alone**. Like for the human network (see Figure 3), the plot reports the new inhibitions that synergisms make possible and the inhibitions with different selectivity with respect to a single drug treatment. Notice how the number of new inhibitions is significantly higher than in the human metabolism, meaning that the cancer pathways are less robust (and redundant) than their counterparts in the human network.
