# Supplementary material for: Predicting and characterizing selective multiple drug treatments for metabolic diseases and cancer
Source: BMC Syst Biol. 2012 Aug 29;6:115. doi: 10.1186/1752-0509-6-115 (PMC3744170; doi:10.1186/1752-0509-6-115)
Supplement: Additional file 3 — Table S3. Cancer vs human network: solutions with an additional target. [file 1752-0509-6-115-S3.pdf]

Table S3 - Cancer vs human network: solutions with an additional target.

Additional targets in boldface are those for which an experimental drug (other than the approved drugs listed in DrugBank) could be found in the literature.

Pathway	*Additional target*	Mimosine (#63)	Canaline (#81)	Lithium (#78)	Dorzolamide (#14)	Trichlormethiazide (#61)	Cerulenin (#62)	Orlistat (#65)	Droxidopa (#24)	Pentoxifylline (#50)	Zonisamide (#54)	Sulfasalazine (#48)	Carmustine (#17) - Fomepizole (#75)	**Rosiglitazone** (#7) - **Cerulenin** (#62)	Rosiglitazone (#7) - Orlistat (#65)	Carmustine (#17) - Cefdinir (#32) - Fomepizole (#75)	Disulfiram (#53) - Mimosine (#63) - Icatibant (#84)
Cysteine met.	Glutathione:cystine oxidoreductase												116			116	
Exchange	Bicarbonate exchange				62	71					247						
	Phosphatidylcholine exchange													**108**	116		
	Glycine exchange																132
	L-Cystine exchange												116				
	L-Tyrosine exchange								86								
	myo-Inositol exchange			43													
	L-Arginine exchange		33														
Fatty acid elong.	**palmitoyl-CoA desaturase**[a]						**47**	**55**									
Folate met.	**methenyltetrahydrofolate cyclohydrolase**[b]	**29**															
Lysine met.	2-Oxoadipate:lipoamide 2-oxidoreductase											138					
Nucleotides	guanylate kinase (GMP:ATP)									62							
	adenylate kinase (d form)									62							
	ribonucleoside-diphosphate reductase (GDP)									62							
	ribonucleoside-diphosphate reductase (ADP)									60							
Oxidative Phosph.	inorganic diphosphatase											317					
R Group Synth.	R group to palmitate conversion													**89**	97		
	R group artificial flux (C16:1)						44	52									
Transport	phosphatidylcholine transporter													**108**	116		
	L-tyrosine transport								86								
Tryptophan met.	2-oxoadipate shuttle											138					
	**3-hydroxyacyl-CoA dehydratase**[c]											**138**					
	**3-hydroxyacyl-CoA dehydrogenase**[c]											**138**					
	**glutaryl-CoA dehydrogenase**[d]											**138**					

[a]: inhibited by 5,6,7,8- tetrahydro-N^5,N^{10}- carbonylfolic acid [49];
[b]: inhibited by isomeric cis-octadecenoic acids [57];
[c]: inhibited by perfluorodecanoic acid [58];
[c]: inhibited by spiropentaneacetic acid [59].
